# Application of tumor pH/hypoxia-responsive nanoparticles for combined photodynamic therapy and hypoxia-activated chemotherapy

**DOI:** 10.3389/fbioe.2023.1197404

**Published:** 2023-06-09

**Authors:** Zhang Zhang, Jintang Feng, Tianzhu Zhang, An Gao, Chunyang Sun

**Affiliations:** ^1^ Department of Radiology and Tianjin Key Laboratory of Functional Imaging, Tianjin Medical University General Hospital, Tianjin, China; ^2^ Department of Radiology, Tianjin Medical University Cancer Institute and Hospital, Tianjin, China; ^3^ Multimodality Preclinical Molecular Imaging Center, Tianjin Medical University General Hospital, Tianjin, China

**Keywords:** tumor pH, responsive nanocarriers, hypoxia-activated prodrug, TAT reactivation, combined therapy

## Abstract

**Introduction:** Cancer selectivity, including targeted internalization and accelerated drug release in tumor cells, remains a major challenge for designing novel stimuli-responsive nanocarriers to promote therapeutic efficacy. The hypoxic microenvironment created by photodynamic therapy (PDT) is believed to play a critical role in chemoresistance.

**Methods:** We construct dual-responsive carriers (^DA^NP_CT_) that encapsulate the photosensitizer chlorin e6 (Ce6) and hypoxia-activated prodrug tirapazamine (TPZ) to enable efficient PDT and PDT-boosted hypoxia-activated chemotherapy.

**Results and discussion:** Due to TAT masking, ^DA^NP_CT_ prolonged payload circulation in the bloodstream, and selective tumor cell uptake occurred via acidity-triggered TAT presentation. PDT was performed with a spatially controlled 660-nm laser to enable precise cell killing and exacerbate hypoxia. Hypoxia-responsive conversion of the hydrophobic NI moiety led to the disassembly of ^DA^NP_CT_, facilitating TPZ release. TPZ was reduced to cytotoxic radicals under hypoxic conditions, contributing to the chemotherapeutic cascade. This work offers a sophisticated strategy for programmed chemo-PDT.

## Introduction

In recent years, a variety of nanocarriers has been designed to deliver therapeutic agents for combined chemo-photodynamic therapy (chemo-PDT) (Kim et al., 2017; Pei et al., 2019; Yu et al., 2023). The complementary cell-killing mechanisms of combined chemo-PDT eliminate the limitations of monotherapy and improve anticancer therapeutic efficacy (Conte et al., 2016; Cheng et al., 2020; Majerník et al., 2022). Despite the theoretical promise of chemo-PDT, outcomes are significantly limited by a lack of precise tumor targeting. Rather than relying on passive enrichment in tumors via enhanced permeability and retention (EPR), tethering cell-penetrating peptides (CPPs) to the nanocarriers promotes payload delivery to tumor cells (Guidotti et al., 2017; Zorko et al., 2022). However, the clearance by macrophages and non-specific distribution in healthy tissue are major obstacles that limit the efficacy of CPPs (Deshpande et al., 2013; Futaki and Nakase, 2017). To overcome these limitations, CPP function must be precisely masked in the bloodstream and activated only within the targeted solid tumors (Dohmen and Wagner., 2011; Zhu et al., 2013). Many studies have focused on developing strategies for spatially controlled, tumor-specific CPP presentation (Huang et al., 2013; Li et al., 2014; Ruoslahti, 2017; Jing et al., 2018; Mohammed et al., 2019). Due to its homogeneity and stability, extracellular acidity (pH_e_ ∼6.5–6.8) is a promising stimulus, and growing evidence has suggested the outstanding sensitivity of the dimethyl maleate (DA) moiety to pH_e_ (Du et al., 2011; Gao et al., 2017; Ma and Sun, 2020).

The photosensitization reaction generally produces many toxic reactive oxygen species (ROS) by consuming surrounding oxygen (Abrahamse and Hamblin, 2016; Han et al., 2022; Tang et al., 2022). As a result, cells that survive PDT exist in a hypoxic microenvironment, and hypoxia-induced chemoresistance has become a critical issue in these residual cells (Wang et al., 2017; Yang et al., 2021). Hypoxia-inducible factor-1α activity is upregulated, altering metabolism and drug efflux in hypoxic tumor cells (Majmundar et al., 2010; Cairns et al., 2011; Ma et al., 2022). Hypoxia also alters DNA methylation and autophagy, which are related to chemotherapy resistance (Jing et al., 2019; Kopecka et al., 2021). Pioneering studies have demonstrated that hypoxia-activated prodrugs (HAPs), which can be converted from non-toxic to toxic molecules under hypoxic conditions, offer a powerful strategy that can be combined with PDT for the selective killing of hypoxic cells after PDT (Feng et al., 2017; Sun et al., 2019; Zhu et al., 2019; Jiang et al., 2022). HAPs typically interact with nuclear DNA, so boosted intracellular drug release is also a highly desirable feature of nanocarriers (Li et al., 2021; Yang et al., 2022). Specific and effective cancer therapy requires the design of nanosystems with pH_e_-sensitive TAT presentation and hypoxia-boosted cargo release to simultaneously achieve tumor homing and HAP liberation and activation inside the targeted tumor.

**FIGURE 1:**
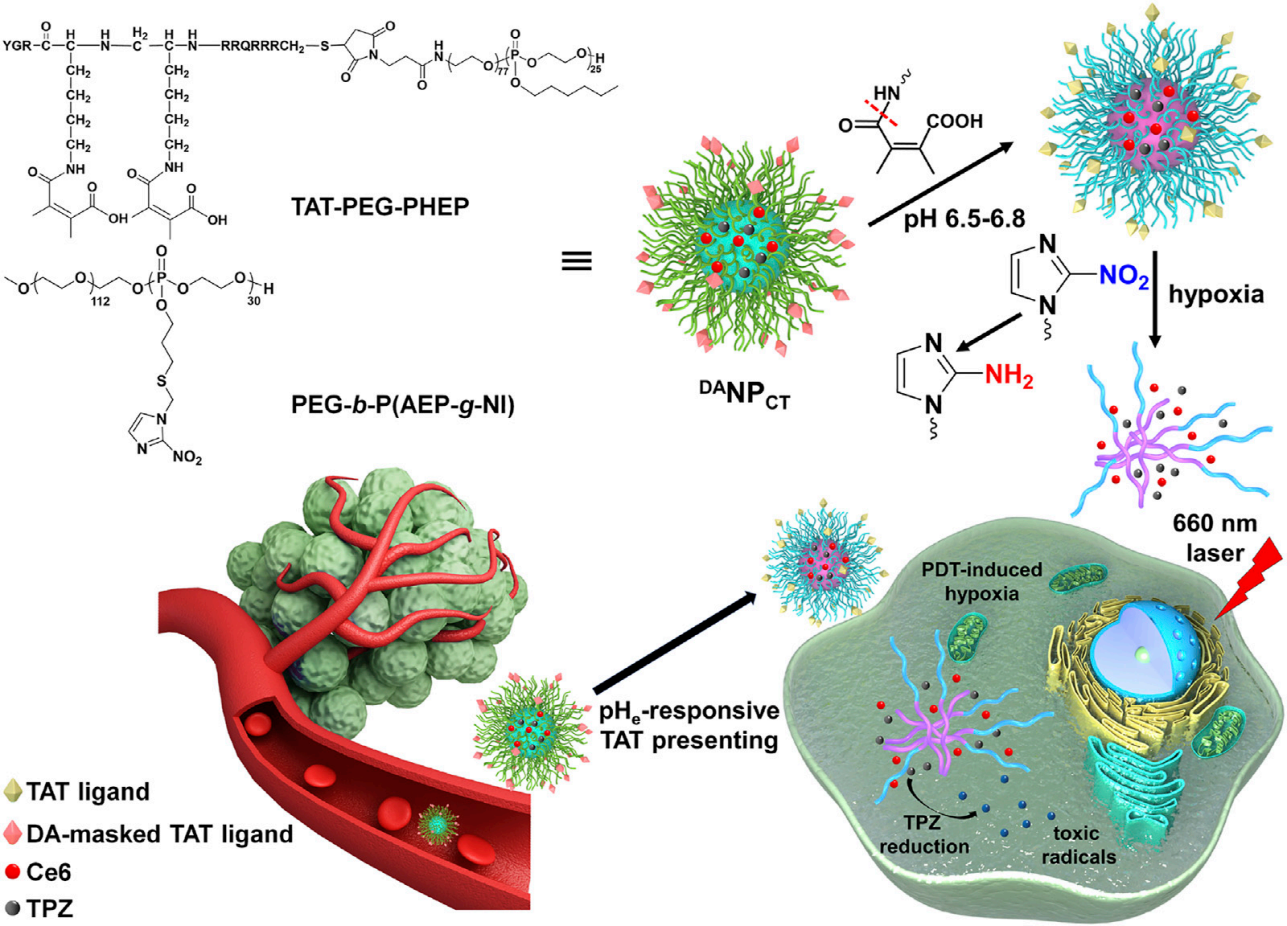
Scheme of ^DA^NP_CT_ design and pH_e_-sensitive TAT presenting and PDT-induced hypoxia-activated chemotherapy.

We developed a mixed polymeric micelle (^DA^NP_CT_) capable of TAT presentation at pH_e_ and hypoxia-responsive dissociation for controlled PDT and hypoxia-activated chemotherapy. TAT-modified poly (ethylene glycol)-polyphosphoesters (TAT-PEG-PHEP) and 2-nitroimidazole-grafted PEGylated polyphosphoesters PEG-*b*-P (AEP-*g*-NI) were self-assembled to encapsulate chlorin e6 (Ce6; photosensitizer) and tirapazamine (TPZ; HAPs). We hypothesized that ^DA^NP_CT_ could achieve prolonged blood circulation due to temporary shielding of the TAT ligands via the DA moiety (Figure 1). After entering the tumor matrix, pH_e_-induced DA deshielding activated the TAT to promote cellular penetration. PDT with a 660-nm laser triggered the production of cytotoxic ROS, thus leading to cell killing and O_2_ consumption. Local hypoxia converted hydrophobic NI to hydrophilic 2-aminoimidazole to facilitate ^DA^NP_CT_ dissociation and TPZ release and activation to produce toxic radical species for the selective killing of hypoxic cells that remain after PDT. Cascade-amplified therapeutic outcomes were studied *in vitro* and *in vivo*.

## Materials and methods

### Materials

Ce6, 2,2-dimethoxy-2-phenyl acetophenone (DMPA), and TPZ were obtained from Macklin. We synthesized (2-nitro-1H-imidazol-1-yl) methanethiol (NI-SH), the diblock copolymer of PEG-*b*-PAEP, and TAT-PEG-PHEP as described previously (Li et al., 2017; Ma and Sun, 2020). The cell counting kit-8 (CCK-8) was obtained from Shanghai Saint-Bio. Dulbecco’s modified Eagle’s medium (DMEM) and fetal bovine serum (FBS) were purchased from Gibco (Gibco, United States). Phalloidin-Alexa Fluor 488 and DAPI were obtained from Beyotime Biotechnology. All other reagents were of analytical grade and used as received.

### Synthesis of diblock PEG-*b*-P (AEP-*g*-NI)

NI-SH (292.8 mg; 1.84 mmol) and DMPA (14.1 mg) were mixed in THF (16 mL) containing 287.2 mg PEG-*b*-PAEP and purged with Ar_2_ for 25 min. The reaction was incubated for 60 min at room temperature under a 365-nm UV light, then transferred to a dialysis tube (MWCO 3500 Da), and dialyzed against ddH_2_O at 4°C. The solution was lyophilized to obtain PEG-*b*-P (AEP-*g*-NI).

### Preparation of Ce6 and TPZ-loaded nanocarriers

TAT-PEG-PHEP, PEG-*b*-P (AEP-*g*-NI), Ce6, and TPZ were mixed at a weight ratio of 3:7:1:1 in DMF. The organic solution was then added slowly to ddH_2_O under gentle stirring. After stirring overnight, DMF and unencapsulated Ce6 and TPZ were removed by dialysis against ddH_2_O. After centrifugation at 800 *g* for 15 min, the nanoparticles (NP_CT_) were collected. To fabricate pH_e_-sensitive ^DA^NP_CT_, NP_CT_ was reacted with excess 2,3-dimethylmaleic anhydride in ddH_2_O at pH 8–9 and 4°C for 6 h and then purified by ultrafiltration. A similar method was used to prepare pH_e_-insensitive ^SA^NP_CT_ with succinic anhydride instead of 2,3-dimethylmaleic anhydride.

### Cellular uptake of nanocarriers at different pH conditions

MCF-7 cells were seeded in 24-well plates and incubated with fresh DMEM containing NP_CT_, ^SA^NP_CT_, or ^DA^NP_CT_ (pretreated at pH 7.4 or 6.5) at 37°C for 6 h. The cells were washed with cold PBS, fixed with paraformaldehyde, and analyzed by FACS. Total protein and Ce6 concentrations in the cell lysate were analyzed using a bicinchoninic acid kit and spectrofluorimetry, respectively.

MCF-7 cells were seeded on coverslips in 12-well plates and incubated with NP_CT_, ^SA^NP_CT_, or ^DA^NP_CT_ (pretreated at pH 7.4 or 6.5) at 37°C for 6 h. The cells were washed with PBS, fixed with 4% paraformaldehyde, and then stained with phalloidin-Alexa Fluor 488 and DAPI, according to standard protocols. The cells were then visualized on a Zeiss LSM 810 confocal laser scanning microscope.

### Cell killing by ^DA^TAT-NP_Ce6_ *in vitro*

To study the biocompatibility of nanoparticles that have not been loaded with Ce6 or TPZ, MCF-7 cells were seeded in 96-well plates (10,000 cells per well) and incubated with NP, ^SA^NP, or ^DA^NP for 72 h. To study the therapeutic efficacy of PDT and hypoxia-activated chemotherapy, MCF-7 cells were seeded in 96-well plates (10,000 cells per well). The normoxic (21% O_2_ pressure) or hypoxic condition (2% O_2_ pressure) was generated in a three-gas incubator, while the partial pressure of CO_2_ was maintained at 5%. NP_CT_, ^SA^NP_CT_, or ^DA^NP_CT_ was added at pH 7.4 or 6.5, incubated for 4 h, and then incubated with MCF-7 cells under normoxic conditions at different Ce6 concentrations for 24 h. The medium was replaced with DMEM (10% FBS) without nanoparticles, and then, the cells were exposed to a 660-nm laser (100 mW/cm^2^; 15 min). After incubation for another 48 h under normoxic or hypoxic conditions, viability was measured using a standard CCK-8 assay.

### Pharmacokinetics and biodistribution of ^DA^NP_CT_ *in vivo*

Female BALB/c mice were randomly divided into four groups and treated with free Ce6, NP_CT_, ^SA^NP_CT_, or ^DA^NP_CT_ via tail vein injection (Ce6 10 mg/kg). At 10 min, 30 min, 1 h, 2 h, 4 h, 8 h, 12 h, 24 h, and 48 h post-injection, blood samples were collected from the retro-orbital plexus. The plasma was obtained by centrifugation, and Ce6 content was quantified by high-performance liquid chromatography (HPLC).

To study the accumulation of ^DA^NP_CT_ in the major organs and tumor tissues, 1 × 10^7^ MCF-7 cell suspension (200 μL) was injected into the mammary fat pad of female BALB/c nude mice to develop a tumor model. Mice bearing MCF-7 xenografts were treated with an intravenous (i.v.) injection of free Ce6, NP_CT_, ^SA^NP_CT_, or ^DA^NP_CT_ (Ce6 10 mg/kg). At 6 h, 12 h, and 24 h, tumor tissues and other organs were excised and homogenized, and Ce6 content was quantified by HPLC.

### Antitumor efficacy *in vivo*

Female MCF-7 tumor-bearing BALB/c nude mice were randomly divided into five groups (n = 6), and, once the tumor volume reached ∼100 mm^3^, the mice were treated with 0.9% NaCl, free Ce6 + TPZ, NP_CT_, ^SA^NP_CT_, or ^DA^NP_CT_ (TPZ 5 mg/kg) every week. At 12 h post-injection, the tumor sites were exposed to 660-nm light for 15 min at a power density of 200 mW/cm^2^. Tumor volume (0.5 × length × width^2^) and body weight were monitored every 3 days. On day 24, blood samples and the major organs were collected for ELISA analysis and hematoxylin and eosin (H&E) staining, respectively.

## Results and discussion

### Preparation of pH_e_- and hypoxia-responsive ^DA^NP_CT_

**FIGURE 2:**
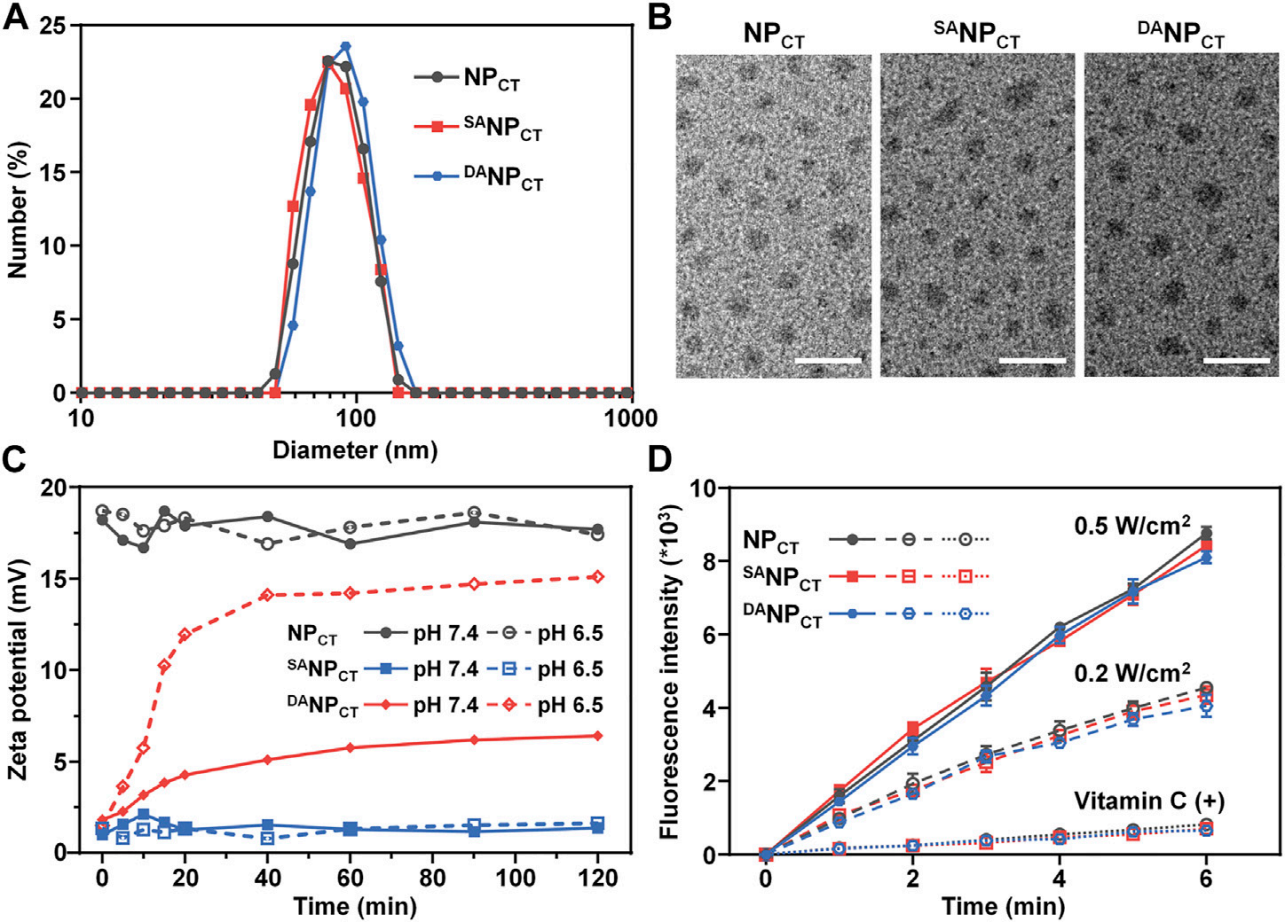
**(A)** Size distribution of NP_CT_, ^SA^NP_CT_, and ^DA^NP_CT_ measured by DLS. **(B)** TEM observation of NP_CT_, ^SA^NP_CT_, and ^DA^NP_CT._ The scale bar is 200 nm. **(C)** Zeta potential change of NP_CT_, ^SA^NP_CT_, and ^DA^NP_CT_ at pH 7.4 or 6.5. **(D)** ROS production indicated by DCFH (E_m_ = 525 nm) of Ce6-loaded nanoparticles. Vitamin C acts as an ROS scavenger.

To prepare hierarchically responsive nanocarriers, TAT-PEG-PHEP and hypoxia-sensitive PEG-PAEP-NI were synthesized as described previously. The efficiency of NI modification was approximately 100% after thiol–ene “click” chemistry, as indicated by ^1^H NMR spectroscopy (Supplementary Figure S1). Hydrophobic Ce6 and TPZ were encapsulated with TAT-PEG-PHEP and PEG-*b*-P (AEP-*g*-NI) at a 3:7 weight ratio to form the mixed micelle (NP_CT_). Finally, 2,3-dimethylmaleic anhydride was introduced to react with the lysine amines of NP_CT_ to yield nanoparticles with pH_e_-responsive TAT reactivation properties (^DA^NP_CT_). For comparison, pH_e_-insensitive ^SA^NP_CT_ was prepared by decoration with succinic anhydride. The hydrodynamic diameter of NP_CT_, ^SA^NP_CT_, and ^DA^NP_CT_ was measured by dynamic light scattering and transmission electron microscopy. The various nanoparticles had a diameter of ∼80 nm and a uniform, spherical morphology (Figures 2A,B). According to the UV-vis absorbance at 642 and 698 nm, the encapsulation efficacies of the mixed micelles were 2.31% (Ce6) and 2.69% (TPZ), respectively. The average size of the nanoparticles, regardless of TAT modification, remained unchanged in PBS over 168 h (Supplementary Figure S2), verifying the outstanding colloidal stability provided by the outer PEG layer.

As expected, the TAT amine groups are exposed to enable cell penetration following degradation at an acidic tumor pH. We monitored the zeta potential at pH 6.5 or 7.4 and found that NP_CT_ maintained its original zeta potential at both pH values, comparable to that of TAT-decorated nanocarriers reported elsewhere (Figure 2C) (Li et al., 2017; Zhang et al., 2020). However, the zeta potential of ^DA^NP_CT_ increased dramatically from +1.4 mV to +15.1 mV at pH 6.5. Meanwhile, the slight zeta potential elevation of ^DA^NP_CT_ at pH 7.4 could be explained as the partial breakage of unstable amide bonds within the DA moieties. With SA modification, ^SA^NP_CT_ masked the TAT ligand, and a minimal zeta potential change was observed at both pH levels. The fluorescamine method was used to quantify the exposed amine groups. Compared to the control groups, the DA degradation efficiency of ^DA^NP_CT_ reached 85.78% ± 4.65% at pH 6.5 (Supplementary Figure S3), and only 22.36% ± 2.58% of DA moieties broke at neutral pH.

The PDT effect at 660 nm was measured using a 2′,7′-dichlorodihydrofluorescein diacetate probe because it was oxidized by ROS to obtain fluorescent DCFH (Eruslanov and Kusmartsev, 2010; Kim and Xue, 2020). Emission fluorescence measurements of NP_CT_, ^SA^NP_CT_, and ^DA^NP_CT_ at 525 nm (excitation = 488 nm) revealed comparable laser power-dependent ROS production rates (Figure 2D). The fluorescence intensity of DCFH induced by NP_CT_, ^SA^NP_CT_, and ^DA^NP_CT_ was reduced by nearly ∼11.6-fold after adding an ROS scavenger (vitamin C). ROS generated by Ce6 was mainly derived from singlet oxygen via the Type 1 mechanism. The abundant ROS generation confirmed that Ce6-encapsulated nanoparticles are efficient for PDT application and accelerated hypoxia-boosted TPZ release.

**FIGURE 3:**
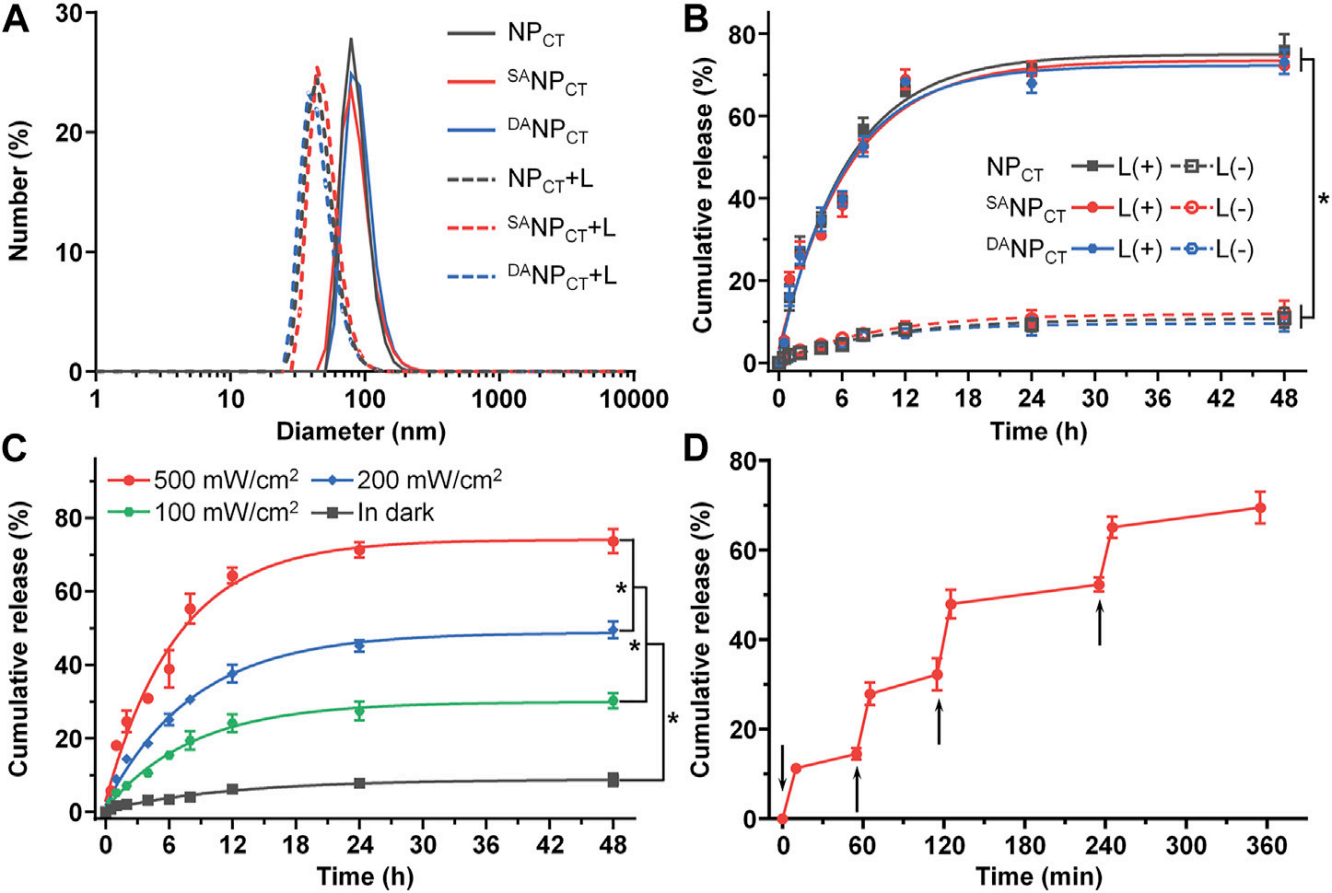
**(A)** Diameter change in NP_CT_, ^SA^NP_CT_, and ^DA^NP_CT_ after exposure to 660-nm near infrared light. **(B)** Cumulative TPZ release profile of NP_CT_, ^SA^NP_CT_, and ^DA^NP_CT_. The power density of 660-nm laser was 500 mW/cm^2^. *p < 0.05. **(C)** Cumulative TPZ release profile of ^DA^NP_CT_ at 500 mW/cm^2^, 200 mW/cm^2^, and 100 mW/cm^2^ or in the dark. *p < 0.05. **(D)** Pulsed 660-nm laser-triggered TPZ release from ^DA^NP_CT_. The samples were exposed to laser at the predetermined time intervals indicated by the black arrows.

According to our design, the hypoxic conditions created by PDT facilitate hydrophobic NI conversion to hydrophilic AI and boost micelle disassembly and cargo release. Therefore, we measured the variability in the diameter of NI-containing nanoparticles after 660-nm laser exposure. The diameters of NP_CT_, ^SA^NP_CT_, and ^DA^NP_CT_ significantly decreased to ∼45 nm following photosensitization (Figure 3A), and negligible changes were observed in the dark. We quantified the TPZ release profiles with or without PDT via fluorescence spectrometry. At 48 h, there were 75.96% ± 3.94%, 72.31% ± 2.14%, and 73.14% ± 2.96% TPZ leakages from NP_CT_, ^SA^NP_CT_, and ^DA^NP_CT_ after laser treatment, respectively (Figure 3B). In contrast, less than 12.50% of the total TPZ was detected without laser irradiation, and there was no significant difference in TPZ release rates. TPZ release from ^DA^NP_CT_ exhibited a power density-dependent pattern, and PDT resulted in 73.66% ± 3.34%, 49.53% ± 2.34%, and 30.21% ± 2.09% of TPZ liberation at different power densities (Figure 3C). The 660-nm laser on/off cycle induced controlled pulses of TPZ release from ^DA^NP_CT_ (Figure 3D). The hierarchy of pH_e_ and hypoxic conditions enabled TAT ligand presentation and cargo release, promoting the accumulation of active drug content at the target site.

#### Cellular uptake of ^DA^NP_CT_ at pH 6.5

**FIGURE 4:**
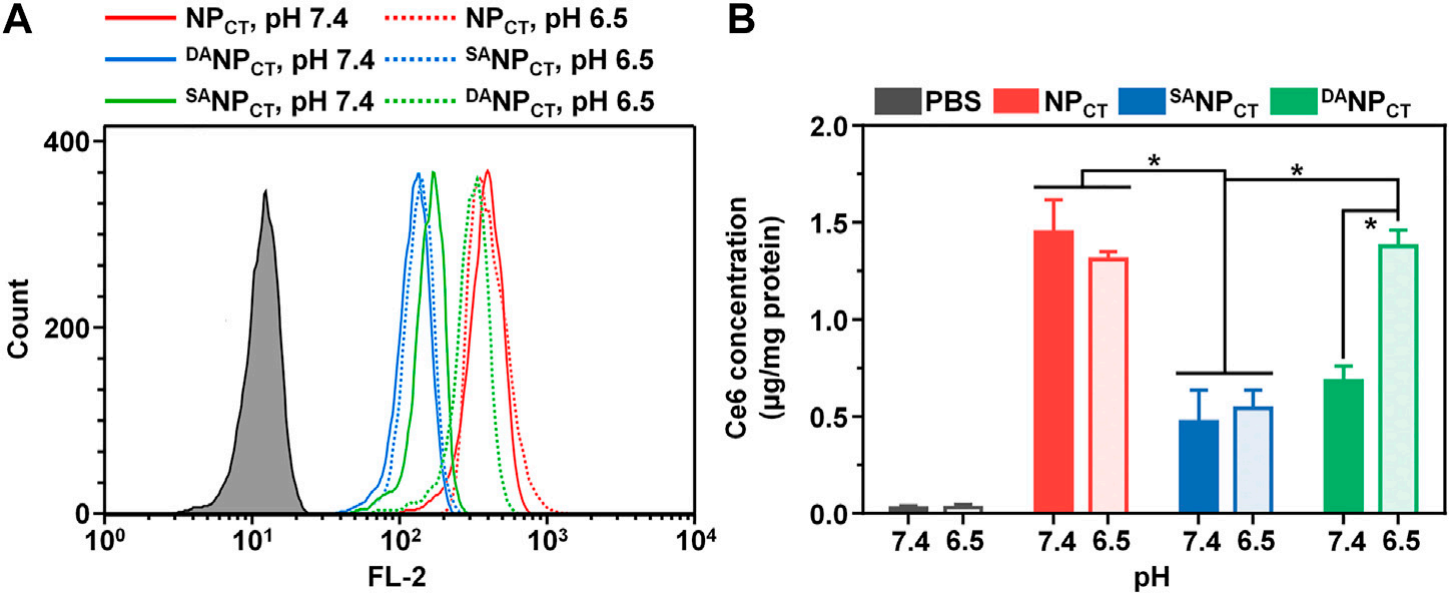
**(A)** Intracellular fluorescence of NP_CT_, ^SA^NP_CT_, and ^DA^NP_CT_ in MCF-7 cells at pH 7.4 or 6.5. **(B)** Intracellular Ce6 concentration in MCF-7 cells at different pH conditions. *p < 0.05.

**FIGURE 5:**
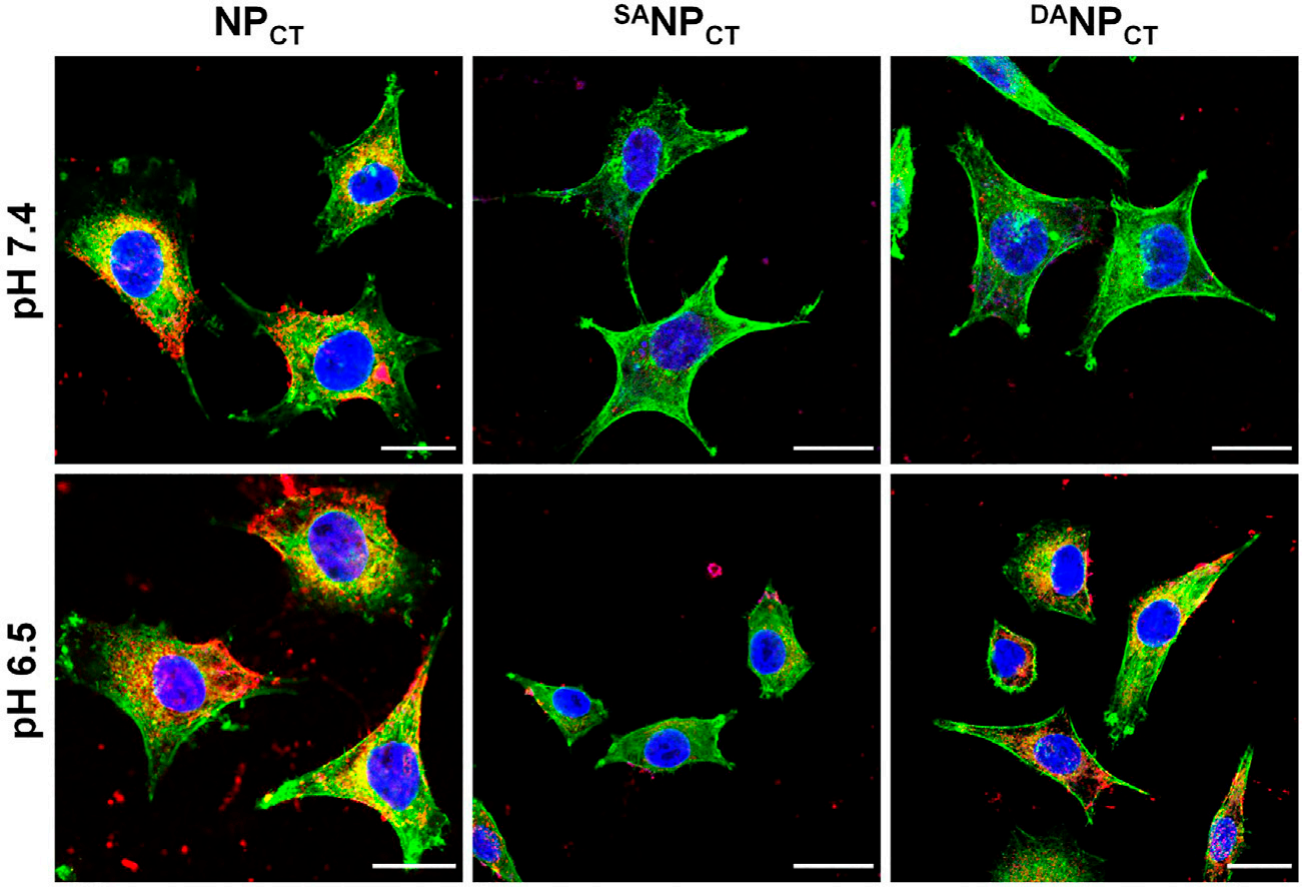
CLSM observation of NP_CT_, ^SA^NP_CT_, and ^DA^NP_CT_ on MCF-7 cells at either pH 7.4 or 6.5. Cell nuclei and F-actin were stained by DAPI (blue) and phalloidin-Alexa Fluor 488 (green), respectively. The scale bar is 20 μm.

To track the TAT presentation of ^DA^NP_CT_ under acidic conditions to facilitate cellular internalization, MCF-7 cells were cultured and treated with NP_CT_, ^SA^NP_CT_, and ^DA^NP_CT_ for 4 h. The internalized NP content was analyzed by FACS, which revealed limited cellular uptake with ^SA^NP_CT_ pretreatment at pH 7.4 and 6.5, suggesting reduced interaction between masked TAT-induced and targeted cells (Figure 4A). Due to the pH_e_-sensitive TAT, the intracellular ^DA^NP_CT_ content was significantly higher at pH 6.5 than at pH 7.4, comparable to that of NP_CT_. Following cell lysis, we used HPLC to detect intracellular TPZ concentration and found that the internalized TPZ of ^DA^NP_CT_ increased from 0.69 ± 0.07 μg/mg protein (pH 7.4) to 1.39 ± 0.09 μg/mg protein (pH 6.5) (Figure 4B). However, there was no noticeable change in the NP_CT_ and ^SA^NP_CT_ groups at neutral or acidic pH. The pH-induced ^DA^NP_CT_ pattern was confirmed by confocal imaging. Compared to the weakened signals in the ^SA^NP_CT_ groups, significantly stronger fluorescence was observed when cells were incubated with ^DA^NP_CT_ pretreated at pH 6.5 (Figure 5). These results verified that the penetration capacity of masked TAT was specifically activated by the extracellular pH microenvironment.

### Cell killing *in vitro*

**FIGURE 6:**
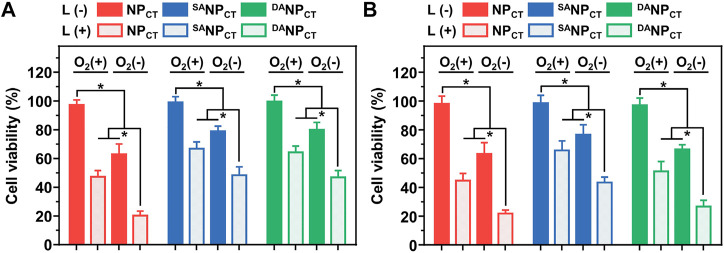
Relative MCF-7 cell viabilities after incubation with NP_CT_, ^SA^NP_CT_, or ^DA^NP_CT_ at pH 7.4 **(A)** or 6.5 **(B)**. *p < 0.05.

The biocompatibility of non-loaded NP, ^SA^NP, and ^DA^NP was evaluated by CCK-8 assay, which showed no notable cytotoxicity in MCF-7 cells at the highest concentration of 500 μg/mL (Supplementary Figure S4). For chemo-PDT effectiveness, cells were incubated with NP_CT_, ^SA^NP_CT_, or ^DA^NP_CT_, exposed to 660-nm light for 20 min, and then cultured at different O_2_ concentrations. Without laser exposure, NP_CT_, ^SA^NP_CT_, and ^DA^NP_CT_ induced low toxicity even under hypoxic conditions because of the absence of the PDT effect and TPZ (HAP) release (Figures 6A,B). However, laser irradiation-triggered cell killing occurred in all groups at comparable levels regardless of pH or O_2_ conditions. Hypoxic culture reduced cell viability, indicating that TPZ was reduced to cytotoxic radicals that interacted with the nuclear DNA. Compared to the viability of MCF-7 cells treated with ^DA^NP_CT_ at pH 7.4, acidic treatment promoted cell killing with 51.07% ± 6.98% cell viability (Ce6 4.0 μg/mL) under normoxic conditions. This difference is attributable to the reactivable TAT ligands and improved Ce6 and TPZ internalization. Under hypoxic conditions, the viabilities of NP_CT_ + L and ^DA^NP_CT_ + L groups at acidic pH were 21.90% ± 2.30% and 26.67% ± 4.29%, respectively, 0.50- and 0.61-fold lower than those of ^SA^NP_CT_ + L. Accordingly, the interactions between PDT and hypoxia-activated chemotherapy indicate that ^DA^NP_CT_ could serve as a robust delivery platform for precise cancer therapy.

### Pharmacokinetic and biodistribution of ^DA^TAT-NP_Ce6_ *in vivo*

**FIGURE 7:**
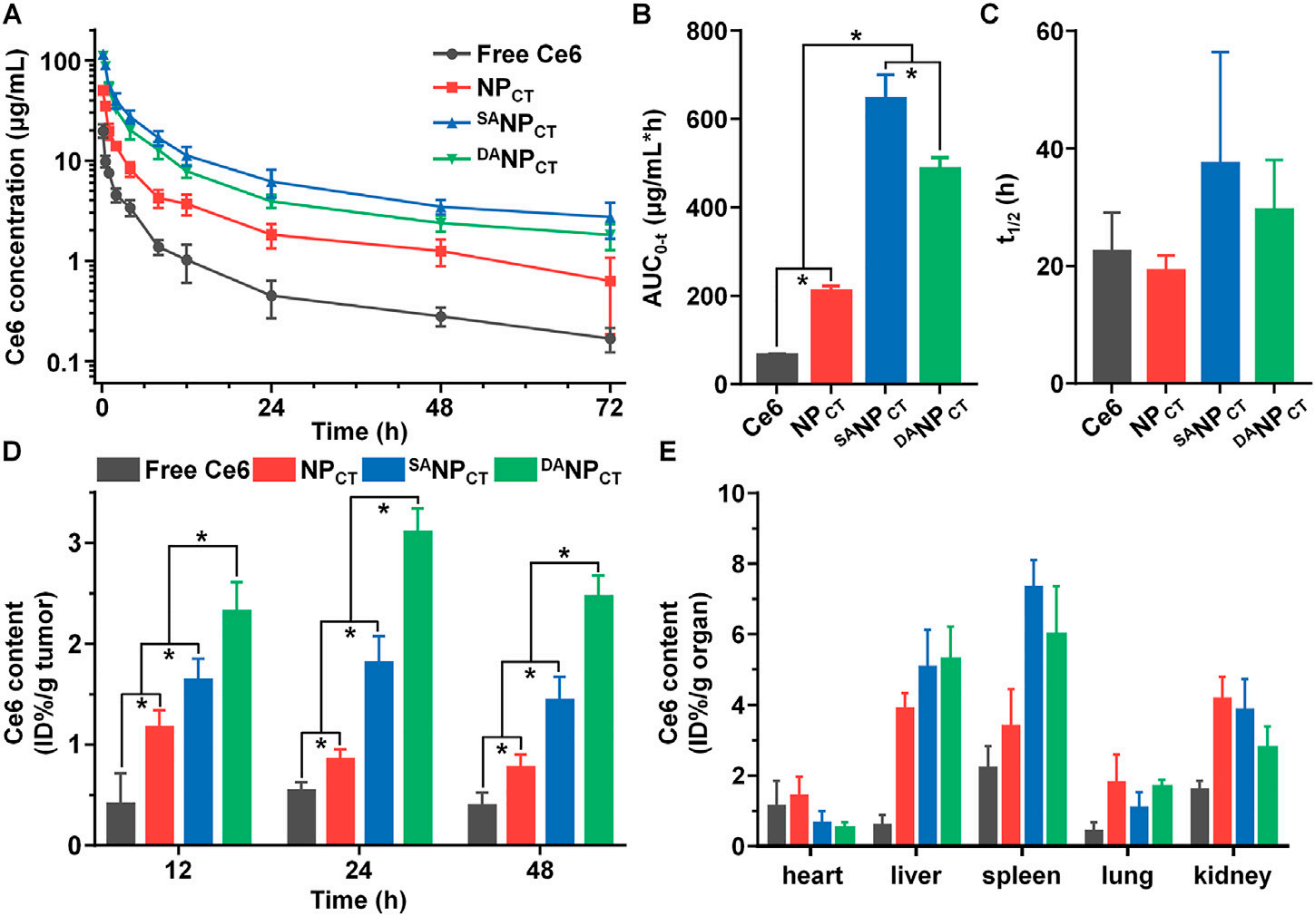
**(A)** Ce6 content in plasma vs*.* time, following *i.v.* injection of free Ce6, NP_CT_, ^SA^NP_CT_, or ^DA^NP_CT_ (n = 4). The area under the curve **(B)** and half-life **(C)** of free Ce6, NP_CT_, ^SA^NP_CT_, or ^DA^NP_CT_ calculated using a non-compartmental model. *p < 0.05. **(D)** Ce6 accumulation in MCF-7 tumor at 12, 24, and 48 h post-injection. *p < 0.05. **(E)** Ce6 distribution in major organs of MCF-7 tumor-bearing mice at 48 h post-injection.

With pH_e_-sensitive DA masking, the penetrating ability of the TAT ligand in ^DA^NP_CT_ was temporally blocked in the bloodstream, limiting phagocytosis. We measured the plasma concentrations of Ce6 following the intravenous injection of different formulations (Figure 7A). NP_CT_ with bare TAT ligands was rapidly cleared from the circulation, yielding a Ce6 concentration of 0.63 ± 0.45 μg/mL at 72 h post-injection. In contrast, both ^SA^NP_CT_ and ^DA^NP_CT_ substantially prolonged the Ce6 circulation, consistent with prior reports (Gao et al., 2017; Zhang et al., 2022). Compared to ^SA^NP_CT_ administration, the Ce6 plasma concentration in the ^DA^NP_CT_ group at 72 h post-injection was reduced from 2.71 ± 1.06 to 1.81 ± 0.54 μg/mL. The pharmacokinetic difference between ^SA^NP_CT_ and ^DA^NP_CT_ could be explained by the slight DA degradation at pH 7.4 (Supplementary Figure S4). We calculated the pharmacokinetic parameters of these nanocarriers using a non-compartmental model (Figures 7B,C) and found that the AUC_0-t_ values of ^SA^NP_CT_ and ^DA^NP_CT_ were 3.08- and 2.32-fold higher than those of NP_CT_, respectively.

Next, NP_CT_, ^SA^NP_CT_, or ^DA^NP_CT_ were *i.v.* injected into BALB/c nude mice bearing MCF-7 xenografts to evaluate their biodistribution, especially toward tumor tissues. After administration, the mice were euthanized at predetermined timepoints, and the Ce6 content in different organs was analyzed by HPLC. ^DA^NP_CT_ had the most preferential retention in tumor tissues at 48 h compared to NP_CT_ and ^SA^NP_CT_ due to the pH_e_-triggered TAT-presenting effect (Figure 7D). Although ^SA^NP_CT_ showed more advanced tumor extravasation via EPR based on the best circulation pattern, stable SA modification impeded TAT ligand interaction and function in the tumor cells, resulting in insufficient tumor accumulation. The amounts of ^DA^NP_CT_ quantified by Ce6 content were 2.32% ± 0.29%, 3.11% ± 0.23%, and 2.47% ± 0.21% ID per gram of tumor at 12, 24, and 48 h, respectively. The nano-sized delivery systems NP_CT_, ^SA^NP_CT_, and ^DA^NP_CT_ accumulated in the liver and spleen, both components of the reticuloendothelial system (Figure 7E).

### Therapeutic efficacy of ^DA^NP_CT_ *in vivo*

**FIGURE 8:**
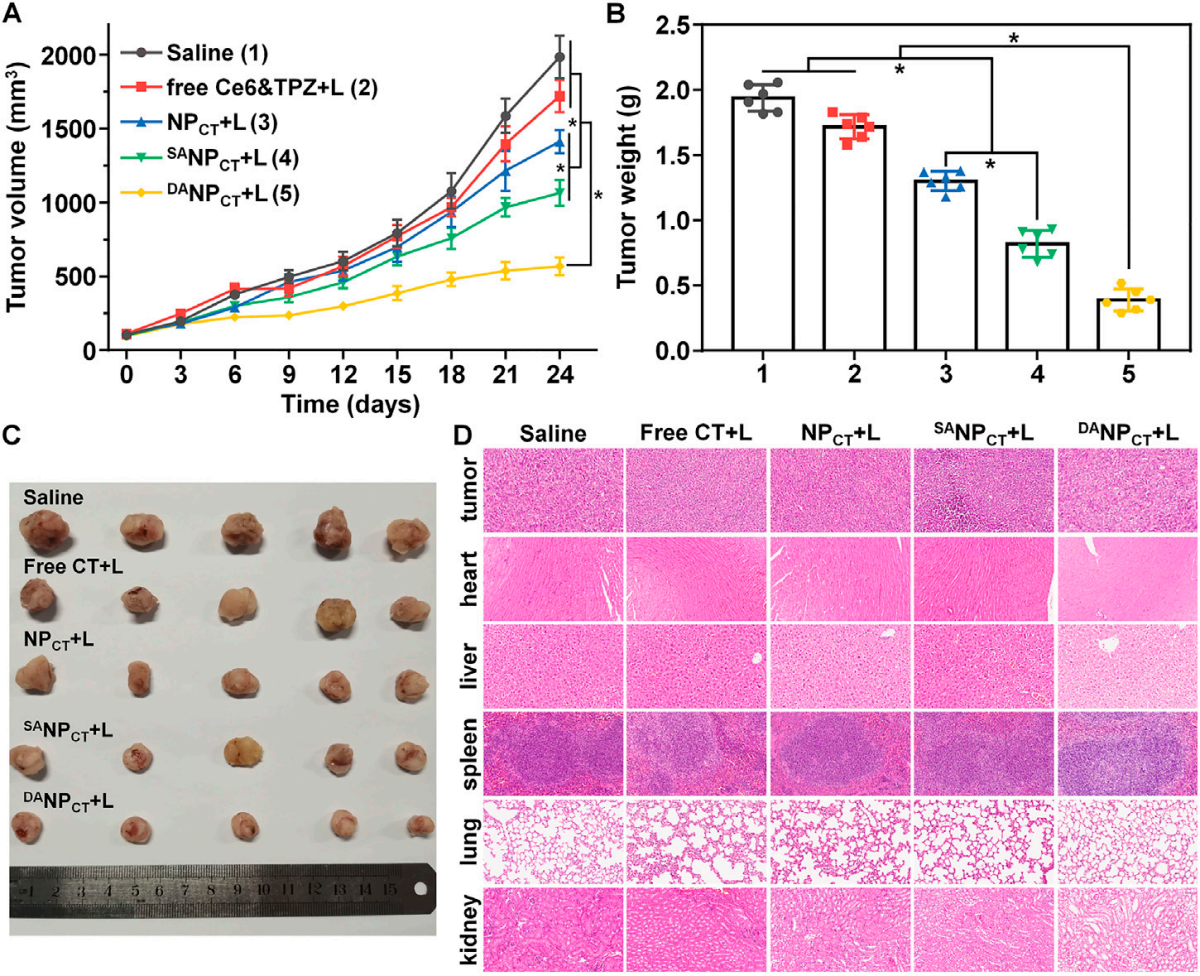
**(A)** Tumor growth curve of the MCF-7 tumor-bearing BALB/c nude mice treated with various formulations. The mice bearing intravenous injections were performed on days 0, 7, 14, and 21. *p < 0.05. **(B)** MCF-7 tumor mass after the treatment. *p < 0.05. **(C)** Tumor images at the end of the treatment. **(D)** H&E staining of tumors and major organs at the end of the tumor treatment.

Encouraged by the excellent *in vitro* performance, we evaluated the *in vivo* antitumor efficacy of ^DA^NP_CT_. MCF-7 tumor-bearing mice were randomly divided and treated via i.v. injection with (1) 0.9% NaCl, (2) free Ce6 + TPZ + PDT, (3) NP_CT_ + PDT, (4) ^SA^NP_CT_ + PDT, and (5) ^DA^NP_CT_ + PDT. The equivalent TPZ dose was 5.0 mg/kg, and the tumor size was recorded every 3 days. Tumor volume in the saline group rapidly increased to 1986.15 mm^3^ at the end of treatment (Figure 8A). Because of their prolonged blood circulation and improved biodistribution, NP_CT_ and ^SA^NP_CT_ significantly inhibited tumor growth after PDT compared to the free Ce6 + TPZ + PDT. Notably, the smallest tumor volumes in the ^DA^NP_CT_ + L group revealed that this treatment yielded the best therapeutic effect, with an average tumor volume of only 567.78 mm^3^ on day 24. The tumor weight after euthanizing confirmed these results (Figure 8B). Tumor mass in the ^DA^NP_CT_ + PDT group was 0.70- and 0.52-fold lower than that in the NP_CT_ + L and ^SA^NP_CT_ + L groups, respectively. In addition, the body weight curves in Supplementary Figure S5 show that the ^DA^NP_CT_ + L group did not experience a decline in the body weight, suggesting the biosafety of ^DA^NP_CT_ *in vivo*. H&E staining (Figure 8C), routine blood count (Supplementary Table S1), and ELISA tests (Supplementary Figure S6) for liver/kidney damage after treatment showed no significant lesions or inflammation in any groups, validating the biocompatibility of the ^DA^NP_CT_ system.

## Conclusion

In this work, a hierarchically responsive nanocarrier ^DA^NP_CT_ was fabricated to spatially control TAT presentation in tumor sites for PDT-initiated, hypoxia-activated cancer therapy. ^DA^NP_CT_ exhibited advanced stability at neutral pH and rapidly reactivated TAT function in response to pH_e_, specifically accelerating internalization by tumor cells. Upon 660-nm laser irradiation, Ce6-based PDT produced cell-killing ROS and consumed surrounding O_2_ to generate hypoxic conditions that stimulated ^DA^NP_CT_ disassembly and TPZ liberation. Chemotherapy with TPZ was enhanced by the aggravated hypoxia in tumor tissues. The biosafety studies of ^DA^NP_CT_ showed excellent *in vivo* biocompatibility with healthy organs. On the other hand, further studies on the feeding ratio of TAT-PEG-*b*-PHEP, PEG-*b*-P (AEP-*g*-NI), Ce6, and TPZ during the preparation of ^DA^NP_CT_ is necessary for optimal therapeutical efficacy *in vivo*. This work contributes to the rational design of tumor microenvironment-responsive nanocarriers for precise and cascade cancer therapies.

## Data Availability

The original contributions presented in the study are included in the article/Supplementary Material; further inquiries can be directed to the corresponding authors.
