# Supplementary material for: Application of tumor pH/hypoxia-responsive nanoparticles for combined photodynamic therapy and hypoxia-activated chemotherapy
Source: Front Bioeng Biotechnol. 2023 Jun 9;11:1197404. doi: 10.3389/fbioe.2023.1197404 (PMC10289258; doi:10.3389/fbioe.2023.1197404)
Supplement: Supplementary file 1 [file DataSheet1.pdf]

Supplementary Material

ROS Generation *in Vitro*.

ROS generation of Ce6-loaded nanoparticles was measured by dichlorofluorescein diacetate (DCFH-DA) as the sensor. DCFH-DA was hydrolyzed to DCFH using NaOH (0.01 M), and then added to free Ce6, NP_{CT}, $^{SA}NP_{CT}$ or $^{DA}NP_{CT}$ in PBS before irradiation, respectively. Following the 660 nm laser irradiation, the DCFH emission fluorescence at 525 nm were recorded by H-7000 fluorescence spectrophotometer (Hitachi, Japan, E_x = 488 nm) to reflect ROS production.

TPZ Release *in Vitro* under 660 nm Laser Activation.

NP_{CT}, $^{SA}NP_{CT}$ or $^{DA}NP_{CT}$ was suspended in phosphate buffer (PB, 20 mM, pH 7.4). The solution was irradiated with 660 nm laser at different power density and then transferred into the dialysis tubing (MWCO 14000 Da), which was immersed in PB at 37 °C with gentle shaking (60 rpm). At predetermined intervals, the external PB buffer was collected and replaced by the fresh buffer. The TPZ content in collected PB was analyzed by UV–vis.

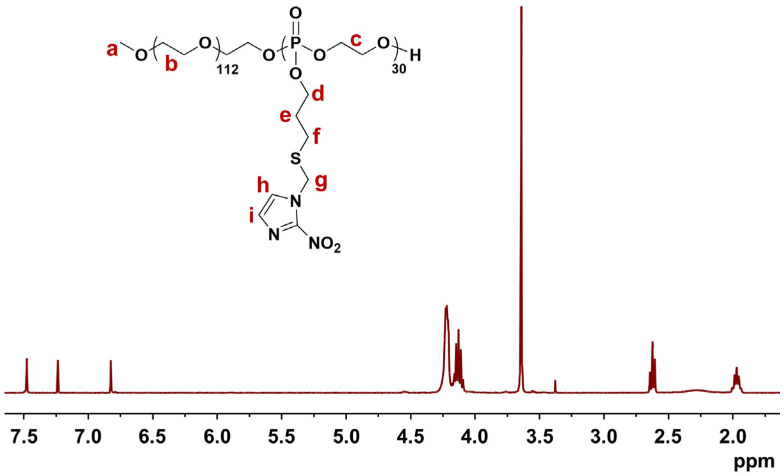

Figure S1. ^{1}H spectrum of PEG-*b*-P(AEP-*g*-NI) in $CDCl_3$.

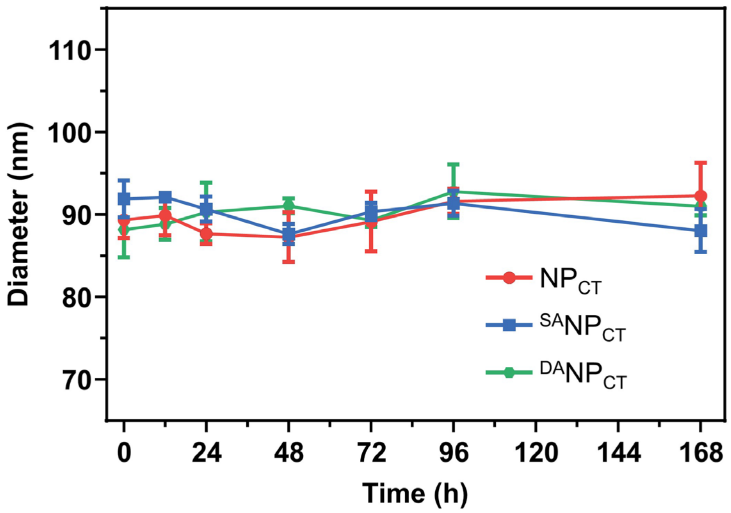

Figure S2. Change of hydrodynamic diameter of NP_{CT}, $^{SA}NP_{CT}$ and $^{DA}NP_{CT}$ in PBS solution.

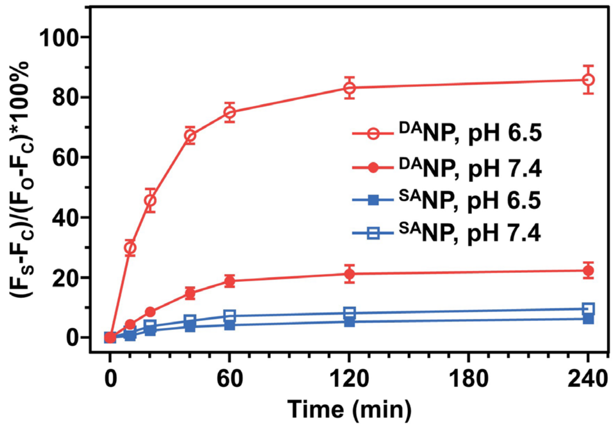

Figure S3. Degradation of nanocarriers using fluorescamine as the sensor. The fluorescence intensity (Fs) of ^{SA}NP or ^{DA}NP was detected (E_x: 390 nm, E_m: 483 nm). F_O was defined as the fluorescence of NP at same concentration. F_C was defined as the fluorescence of PBS control.

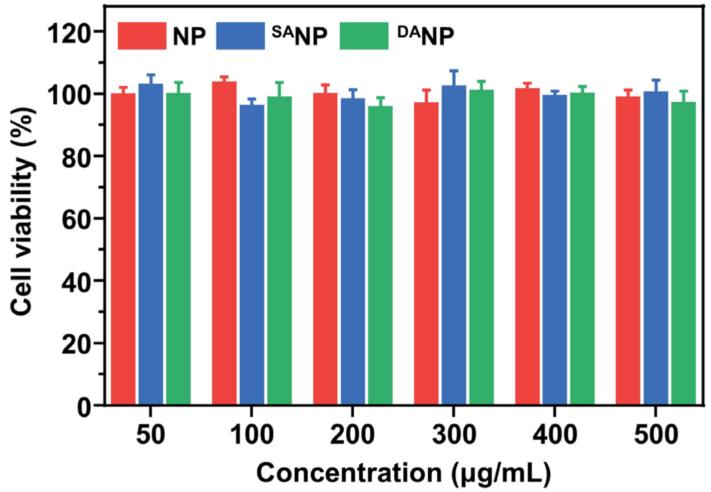

Figure S4. Cell viabilities of MCF-7 cells incubated with nanoparticles without Ce6&TPZ encapsulation.

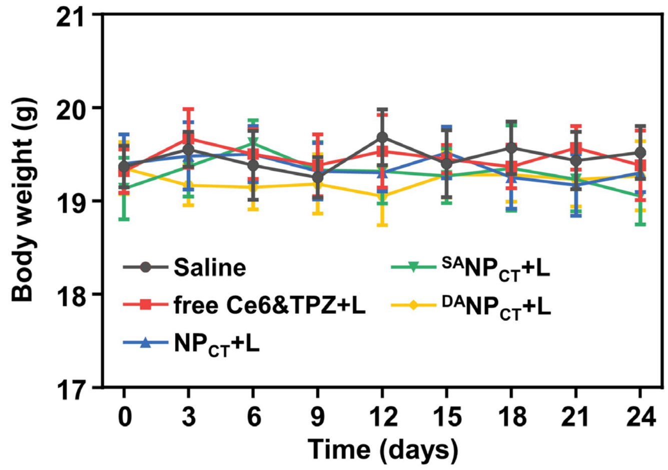

Figure S5. Body weight change of MCF-7 tumor-bearing mice treated with various formulations.

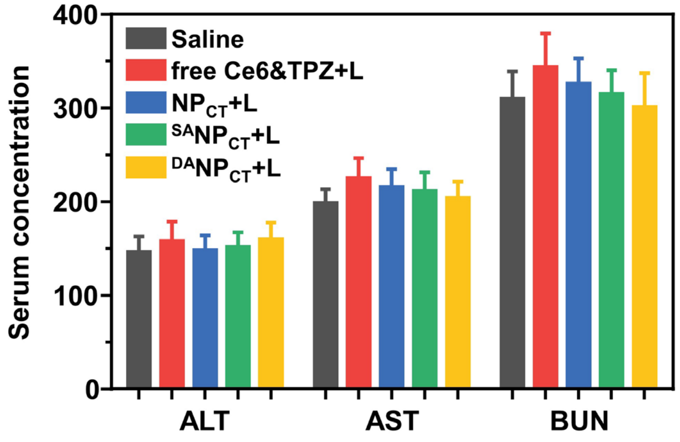

Figure S6. ELISA examination of ALT (U/L), AST (U/L) and BUN (10 nmol/mL) in the serum on day 24.

	Saline	Free Ce6&TPZ	NP_{CT}+L	$^{SA}NP_{CT}$+L	$^{DA}NP_{CT}$+L
WBC	4.54±0.56	5.03±0.73	4.91±0.25	4.82±0.70	5.06±0.61
RBC	9.57±0.27	9.38±0.18	9.42±0.29	9.56±0.20	9.36±0.19
HGB	161.2±3.92	163.8±5.42	164.8±3.92	163.5±2.74	162.0±5.06
HCT	33.73±2.04	32.05±0.48	32.52±0.86	34.22±0.62	35.01±1.14
MCV	35.28±2.73	34.18±1.05	34.54±1.43	35.77±1.05	37.42±1.18
MCH	16.45±0.37	16.08±0.51	16.32±0.30	15.98±0.43	16.23±0.26

Supplementary Table S1. Routine blood analysis of BALB/c mice treated with different formulations. WBC: white blood cells, RBC: red blood cells, HGB: hemoglobin, HCT: hematocrit, MCV: mean corpuscular volume, MCH: mean corpuscular hemoglobin.
